# Postdiction: When Temporal Regularity Drives Space Perception through Prestimulus Alpha Oscillations

**DOI:** 10.1523/ENEURO.0030-21.2021

**Published:** 2021-09-07

**Authors:** Laetitia Grabot, Christoph Kayser, Virginie van Wassenhove

**Affiliations:** 1 Cognitive Neuroimaging Unit, NeuroSpin, CEA, INSERM, CNRS, Université Paris-Saclay, Gif-sur-Yvette 91190, France; 2 Department for Cognitive Neuroscience, Faculty of Biology & Cognitive Interaction Technology, Center of Excellence, Bielefeld University, 33615 Bielefeld, Germany

**Keywords:** alpha oscillations, MEG-EEG, postdiction, rabbit illusion, retroperception, spatiotemporal illusion

## Abstract

In postdiction, the last stimulus of a sequence changes the perception of the preceding stimuli. Postdiction has been reported in all sensory modalities, but its neural underpinnings remain poorly understood. In the rabbit illusion, a sequence of nonequidistant stimuli presented isochronously is perceived as equidistantly spaced. This illusion might be driven by an internal prior favoring a constant-speed motion. Here, we hypothesized that prestimulus alpha oscillations (8–12 Hz), known to correlate with perceptual expectations and biases, would reflect the degree to which perceptual reports are influenced by a constant-speed prior. Human participants were presented with ambiguous visual sequences while being recorded simultaneously with MEG and EEG: the same sequences yielded an illusory perception in about half the trials, allowing contrasting brain responses elicited by identical sequences causing distinct percepts. As a proxy of an individual’s prior, we used the percentage of perceived illusion and the detection criterion, assuming that a strong constant-speed prior would result in a higher rate of illusory percepts. We found that high frontoparietal alpha power was associated with perceiving the sequence according to the individual’s prior: participants with high susceptibility to the illusion would report the illusion, while participants with low susceptibility would report the veridical sequence. Additionally, we found that prestimulus alpha phase in occipitoparietal regions dissociated illusion from no-illusion trials. We interpret our results as suggesting that alpha power reflects an individual’s constant-speed prior, whereas alpha phase modulates sensory uncertainty.

## Significance Statement

Late events may influence how earlier events are perceived, as if the arrow of time was reversed in the brain. This surprising phenomenon, called postdiction, is observed in the rabbit illusion and highlights a predominant mechanism for perceptual processes. Perception builds on the combination of prior expectations with incoming sensory evidence, which takes time. We show that prestimulus neural activity, and more specifically alpha oscillations (8–12 Hz), play a dual role in postdiction: the power of frontoparietal alpha reflects an individual’s prior expectation, whereas occipitoparietal alpha phase predicts illusory perception. Postdiction may be a means to compensate for the neural delays inherent to perceptual processing, so that the arrow of perceptual time matches the arrow of physical time.

## Introduction

Our phenomenological awareness of the world results from the comparison between incoming sensory inputs unfolding over time, and the predictions of our internal models. Predictive models explicitly rely on the serial unfolding of events in time and do not currently accommodate how, over a few tens of milliseconds, later events can modify the perception of earlier events. In the rabbit illusion, sequential stimuli can causally affect the perceived localization of earlier events (Geldard and Sherrick, 1972): when three flashes are presented at a regular rate (isochronously) in the visual periphery, with the first two flashes at the same location and the last one at a different location (nonequidistantly), participants often mislocalize the second flash to be in between the first and the last flash. This illusion has been argued to instantiate a true temporal illusion (Dennett and Kinsbourne, 1992) and to provide the means to study postdiction (Eagleman and Sejnowski, 2000), which may constitute a ubiquitous, yet largely underrated mechanism shaping perception at large (Shimojo, 2014).

Computational and behavioral studies of the rabbit illusion proposed that the spatial distortion induced by the temporal structure of events could be driven by internal priors that favor constant-speed motion (Jones and Huang, 1982; Goldreich and Tong, 2013; Khoei et al., 2017). The expectation that a stimulus maintains a slow and constant speed, combined with isochronous sensory evidence and the spatial uncertainty about the position of an event would result in spatial equidistance in perception.

Despite a large body of psychophysical work, only two neuroimaging studies have started investigated the neural correlates of postdiction (Blankenburg et al., 2006; Stogbauer et al., 2007), and none has addressed this working hypothesis, leaving clear gaps in our understanding of the neural mechanisms underlying postdiction. Here, our aim was to investigate the neural processes capturing the impact of priors in postdiction by recording human brain activity with simultaneous magnetoencephalography (MEG) and electroencephalography (EEG). We tested participants on visual sequences that were tailored so that the same isochronous visual sequence elicited the rabbit illusion half of the time. To measure the influence of constant-speed priors, we used two behavioral parameters measured for each participant: their percentage of illusory percepts and their detection criterion. These two variables were taken as measures of perceptual biases, which may be induced by the constant-speed prior involved in postdiction, with a strong prior resulting in a larger percentage of illusory trials.

Neurophysiologically, alpha rhythms (8–12 Hz) may reflect internal priors and carry perceptual expectations (Mayer et al., 2016; Samaha et al., 2018). Many studies have shown that alpha oscillations correlate with the perception of upcoming stimuli (Ergenoglu et al., 2004; Hanslmayr et al., 2007; van Dijk et al., 2008; Mathewson et al., 2009), but, quite interestingly, rather than impacting accuracy, alpha oscillations seem related to perceptual biases (Iemi et al., 2017) and subjective measures of confidence (Samaha et al., 2017; Wöstmann et al., 2018) or awareness (Lange et al., 2013; Benwell et al., 2017; Gulbinaite et al., 2017). In line with these observations, recent work has shown that on a per individual basis, an increase of prestimulus alpha power is associated with the stronger influence of a participant’s idiosyncratic biases during the processing of temporal sequences (Grabot et al., 2017; Grabot and Kayser, 2020).

Considering that perceptual biases may arise from individual’s prior expectations (de Lange et al., 2018), we hypothesized that the power of the prestimulus alpha power would reflect how much an individual’s prior is involved in postdiction. We tested this working hypothesis by using a between-participant design and by correlating the individual’s fluctuations of prestimulus alpha power with the two behavioral measures capturing the individual’s internal prior. Second, we tested the alternative working hypothesis in which alpha power would predict the accuracy of perceptual reports (Ergenoglu et al., 2004; Mathewson et al., 2009), by contrasting prestimulus alpha power between trials in which participants reported perceiving the illusion or not (illusion and no-illusion trials, respectively). To complement these analyses, we investigated whether the phase of low-frequency oscillations could dissociate illusion from the no-illusion trials. The phase of low-frequency oscillations has previously been reported to influence the perception of upcoming stimuli (Busch et al., 2009; Mathewson et al., 2009) and illusory perception more generally (Dugué et al., 2011; Chakravarthi and VanRullen, 2012; Lange et al., 2014; Samaha and Postle, 2015). Given the literature (VanRullen, 2016b) and the timing (<200 ms) of visual sequences used to elicit postdiction (Geldard, 1982), we focused on the alpha (8–12 Hz) and the theta (4–7 Hz) bands.

## Materials and Methods

### Participants

Nineteen right-handed participants (8 males; age, 28 years; SD, 6) took part in the study. All had normal or corrected-to-normal vision, none had a known neurologic or psychiatric history, and none were under medical treatment. All participants were naive as to the purpose of the study. Each participant provided a written informed consent in accordance with the Declaration of Helsinki (2013) and the Ethics Committee on Human Research at NeuroSpin (Gif-sur-Yvette, France). Six participants were excluded a priori from the analysis because of the failure of the MEG digitization procedure (one participant), MEG data that were too noisy (one participant), behavioral performance below the 60% criterion in the control task (for one or two sequence directions; three participants), and an unbalanced number of illusion and no-illusion trials (one participant). Hence, a total of 13 participants (6 males; age, 29 years; SD, 7) were considered for the MEG-EEG analyses.

### Stimuli

The visual stimuli were M-scaled 2D Gaussian blobs presented as an isochronous sequence of three flashes on a black background. The visual sequence of flashes was always located on the right horizontal meridian. The three possible flash locations were at 15.1°, 18.5°, and 21.8° of visual angle. To account for visual acuity differences according to the stimulus position to fovea (Duncan and Boynton, 2003), the diameter of each flash increased with eccentricity, yielding flashes of 2.5°, 3°, and 3.5° of visual angle, respectively.

**Figure 1.**
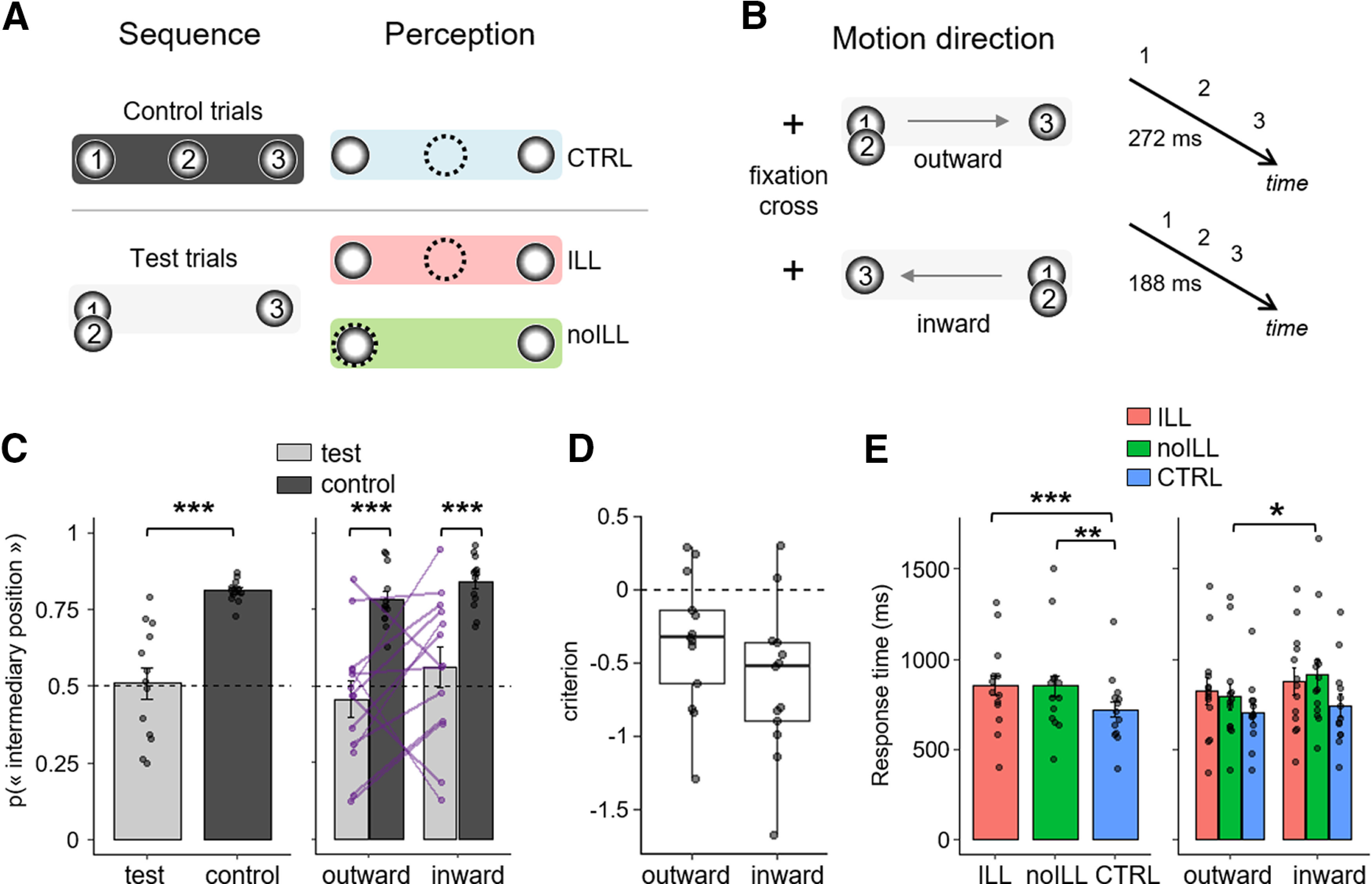
Experimental design and behavioral results. ***A***, In test trials, the two first flashes of the sequence were presented at the same spatial location. The second flash was perceived correctly (no-ILL, green) or at an intermediary position (ILL, red). In control trials, the second stimulus was presented and perceived at an intermediary position (CTRL; in blue). Numbers in the circles indicate the temporal order of each flash within the sequence; dashed circle indicates the perceived position of the second flash. ***B***, All stimuli were presented on the right horizontal meridian. Two directions for the sequence were possible, to ensure a balanced focus of spatial attention (outward or inward). The SOA was equal to 272 ms in outward condition and 188 ms in inward condition. ***C***, Left, The probability of perceiving the intermediary position in test trials (light gray) and control trials (dark gray) in participants. Right, The same data split following on direction conditions. The purple lines connect individual percentages of illusion in test trials between conditions. The dotted line corresponds to 50% of the intermediary position response. ***D***, The boxplots represent the criterion for each sequence direction (median, 25% and 75% quantiles, with individual data points). ***E***, Left, response times (computed from the onset of the last stimulus) were faster in CTRL trials (blue) than in test trials (ILL, red, and no-ILL, green). Right, Response time is faster in the outward than the inward condition. ***p < 0.001, **p < 0.01, *p < 0.05.

All sequences could be presented in an outward (centrifugal) or in an inward (centripetal) motion relative to the fixation dot (Fig. 1*B*). The two motions were included to ensure that participants paid equal attention to all three possible spatial locations (Kilgard and Merzenich, 1995). As the direction of motion was equal across sequences, participants could not predict where the initial flash for the next sequence would start.

Importantly, we tested two types of sequences in both inward and outward motions, which constituted our main experimental conditions: the control and the test sequences. In the control sequences, flashes were regularly spaced (equidistant) so that a flash was presented in each possible location and in a smooth motion; hence, the second flash was effectively presented at an intermediate spatial location between the first and the last flash in a given sequence, regardless of it being inward or outward going. The test sequences were designed to elicit the rabbit illusion: for this, the second flash was presented at the same location as the first flash in the sequence (Fig. 1*A*) for both inward and outward motions. In all sequences, the second flash was slightly brighter than the other two flashes (contrast of 1 relative to the black background, compared with 0.4 for the first and last flashes). All flashes were presented for 50 ms, and their stimulus onset asynchrony (SOA) was constant, yielding an isochronous temporal sequence of flashes. The SOAs were determined in a pilot study (*N* = 5 participants) and selected such that participants reported a rabbit illusion in half of the test trials. For the inward test and control sequences, the SOA was thus set to 188 ms. For the outward test and control sequences, the SOA was set to 272 ms. The causes for the temporal asymmetry between centripetal and centrifugal timings are unclear, but they are consistent with the spatial asymmetry observed in the related flash-lag temporal illusion (Kanai et al., 2004).

### Experimental procedure

Before the MEG-EEG recordings, participants were acquainted with the task for a few minutes. Their task consisted in reporting in a two-alternative forced choice (2-AFC) whether the second flash was (or was not) in an intermediate position between the first and last flashes. The locations of the first and the last flashes in each sequence were fixed. Participants were asked to fixate a green dot located in the middle of the screen during the whole experiment. Participants answered using their right index and middle fingers on two buttons of a fiber optic response pad (Science Plus Group; red button, “yes”; green button, “no”).

The intertrial interval (ITI) was randomly drawn from a uniform distribution between 800 and 1300 ms following the participant’s response. This was done to prevent the use of a rhythmic or supratemporal strategy to solve the task given that visual sequences were isochronous, and the random ITI clearly delineated sequential processes per single trial.

The experiment was divided into five blocks of ∼8 min each. One experimental block tested 120 trials of each test condition (60 trials inward, 60 trials outward) and 60 trials of each control condition (30 trials inward, 30 trials outward). The order of presentation was randomized both within participants and across participants. Each test condition was thus repeated for a total of 600 times (300 trials inward, 300 trials outward); each control condition was repeated for a total of 300 times (150 trials inward, 150 trials outward). A total of 900 trials was collected per participant.

### Analysis of behavioral data

The response times (RTs) were computed from the onset of the last flash in the sequence. Trials with RTs <0.2 or >4 s from the offset of the second stimulus were rejected (rejection rate, ∼2%). The statistical analyses were conducted with the R suite (2013 version; R Foundation for Statistical Computing). A two-way repeated-measures ANOVA was conducted on the probability of perceiving the second flash in an intermediary position with factors of condition (two: test, control) and sequence direction (two: inward, outward). RTs were analyzed using a two-way repeated-measures ANOVA with factors of perceptual report (three: illusion, no-illusion, control) and sequence direction (two: inward, outward). *Post hoc* Bonferroni-corrected paired *t* tests were computed as well as the Bayes factor (BF) for paired *t* tests (*BayesFactor* package with R).

Following standard detection theory analysis (Green and Swets, 1966), we estimated for each individual the criterion (*c*) based on an adapted confusion matrix where the hit rate (*H*) corresponds to the percentage of control trials correctly perceived and the false alarm (FA) rate corresponds to the percentage of test trials perceived as illusory, as follows:

$$c=-\frac{z\left(H\right)+z(FA)}{2}.$$

### Combined MEG-EEG and anatomic MRI data acquisition

Electromagnetic brain activity was simultaneously recorded with MEG-EEG in a magnetically shielded room. The MEG data were collected using a whole-head Elekta Neuromag Vector View 306 MEG system (Neuromag Elekta) in an upright position, a system equipped with 102 triple sensor elements (one magnetometer and two orthogonal planar gradiometers). The EEG was recorded using an Easycap EEG cap (60 electrodes). The MEG-EEG data were collected at 1 kHz sampling frequency with a high-pass filter of 0.3 Hz. Horizontal, vertical electro-oculogram (EOG; four electrodes) and electrocardiogram (EKG; three electrodes) were collected with typical electrode placements during the session. The reference electrode for the EEG was placed on the tip of the participant’s nose, and data were rereferenced to a common average in subsequent analyses.

The participant’s head position was measured before each block using four head position coils (HPI) placed over the frontal and mastoid areas. The HPI, fiducial points, and EEG electrodes were digitalized using a 3D digitizer (Polhemus) to help with the coregistration with anatomic MRI. Anatomical landmarks were used to locate the fiducial points (nasion and the left and right preauricular areas) and to coregister electrophysiological recordings with anatomic MRI data. Subsequent to the MEG-EEG experiment, anatomic MRI images were obtained using a 3 T Trio MRI Scanner (Siemens). An MPRAGE sequence was used as well as the multiecho FLASH (fast low-angle shot) sequence with flip angles at 5° and 30°, which ensure a better separation for skin and skull boundaries during the creation of the head model.

### MEG-EEG data preprocessing

First, signal space separation (SSS) was applied to the raw continuous data to decrease the impact of external noise (Taulu and Kajola, 2005). The SSS correction, the head movement compensation, and the noisy MEG sensors and EEG electrodes interpolation were applied using titrated parameters of the MaxFilter Software (Elekta Neuromag) and visual inspection of the raw data.

The second step of MEG-EEG data preprocessing included an ocular and cardiac artifacts reduction using independent component analysis (ICA) decomposition. MEG-EEG data were aligned to the detected EKG and EOG peaks directly recorded with EKG and EOG, respectively. The ICA components capturing ocular and cardiac artifacts in brain responses were computed on these time-aligned signals separately for EEG and MEG recordings. The correction consisted in rejecting ICA components of brain data most correlated with the EKG and EOG signals. The criterion was defined by a Pearson correlation score between MEG-EEG data and the EKG and EOG signals with an adaptive *z*-scoring (threshold, 3). All outcomes were verified by visual inspection.

Following the artifact reduction procedure, a final cleaning step consisted in rejecting an individual’s epoch when its amplitude exceeded a fixed threshold (gradiometers, 4000 e^−13^ T/m; magnetometers, 40 e^−13^ T; EEG, 250 e^−6^ V). The outcome of this procedure was also verified by visual inspection.

### Analysis of MEG-EEG data

MEG-EEG data analysis was performed with MNE-Python (Gramfort et al., 2014), MATLAB version R2017a (MathWorks) and the Fieldtrip toolbox (Oostenveld et al., 2011). The trials were classified as a function of the participant’s behavior, and only correct trials were considered for the analysis of the control condition; in the test trials, we separated trials perceived as an illusion (illusion condition, or ILL) from those in which the correct sequence was reported (no-illusion condition, or no-ILL). Raw data were epoched relative to the onset of the first stimulus in the sequence. All epochs were 2 s long from –0.8 s before the first stimulus of the sequence to +1.2 s. All epochs were downsampled to 333 Hz and low-passed filtered at 160 Hz using IIR forward–backward filtering. The number of trials was equalized as a function of the responses (illusion or no-illusion) and for each direction. On average across participants, 94 ± 36 and 74 ± 25 trials (mean ± SD) were used per response, for the outward and inward direction, respectively.

#### Power analysis

Single-trial time–frequency analysis was performed on the MEG-EEG data for each sensor type. The power and the intertrial phase coherence (ITC) were calculated using Morlet wavelets with a fixed 3 Hz bandwidth (corresponding to 3.7 cycles at 5.5 Hz, 6.7 cycles at 10 Hz, 14.3 cycles at 21.5 Hz, 35 cycles at 37.5 Hz, and 53.3 cycles at 80 Hz). The resulting power and ITC were averaged for each frequency band of interest (theta, 4–7 Hz; alpha, 8–12 Hz; beta, 13–30 Hz; low-gamma, 30–45 Hz; high gamma, 60–100 Hz). As our working hypothesis pertained to the prestimulus activity in the alpha band, no baseline normalization was applied (Hanslmayr et al., 2007; Busch et al., 2009; Jensen and Mazaheri, 2010). Alpha power was computed for each inward and outward direction separately. We restrained the prestimulus period from −600 to −200 ms to avoid edge effects because of the time–frequency analysis and the contamination of poststimulus responses. The power on planar gradiometers was combined using Euclidean norm.

Statistical testing relied on a spatiotemporal clustering procedure based on a permutation *t* test, performed for each sensor type with MNE-Python (cluster-forming threshold, *p* < 0.01, adjusted for two-sided test) to contrast ILL and no-ILL trials for combined inward and outward direction. Only clusters with a *p* value <0.05 were reported. We also correlated the difference in prestimulus alpha power between ILL and no-ILL trials, and the individual percentage of illusory perception or the individual criterion, for each time point and sensors. The statistical testing again relied on a spatiotemporal cluster-based permutation test obtained using Fieldtrip functions (cluster threshold, 0.01, two-sided test; minimum neighbors, 2; 4000 repetitions) for each sensor type. The permutation distribution was obtained by shuffling the participants’ labels. We calculated the Spearman’s correlation coefficient on data averaged on the clusters indicating a significant difference, and reported percentile-bootstrapped 95% confidence intervals (CIs; 2000 repetitions). We also reported the Bayes factor for Pearson correlation using the *BayesFactor* toolbox in MATLAB, and interpreted the Bayes factor following the procedure in the study by Jarosz and Wiley (2014).

Since the results showed a significant correlation in each direction, we then investigated whether this effect was localized in sensors common to both directions. To address this question of a common localization, we ran a conjunction analysis between directions, which consisted, for each time point and sensor, in keeping the lowest absolute value of correlation coefficient between directions. The conjunction R was then compared with a null distribution obtained by shuffling individuals (4000 repetitions) using spatiotemporal clustering to correct for multiple comparisons (cluster threshold, 0.01; using the percentiles of the null distribution, adjusted for two-sided test, with cluster statistics being the maximum over cluster).

#### Phase analysis

We used the phase opposition sum (POS; VanRullen, 2016a) to investigate whether the phase of prestimulus alpha activity correlated with the perception of test trials as illusory or veridical. The POS is based on the ITC measured over trials for each condition separately, here illusion and no-illusion (ITC_ILL_ and ITC_no-ILL_, respectively) and over all trials (ITC_all_): POS = ITC_ill_ + ITC_noill_ – 2*ITC_all_.

To test the statistical significance of the POS, we used a permutation procedure (VanRullen, 2016a) on the prestimulus period (−600 to −200 ms). We first built a null distribution for each individual by shuffling the trial labels and recomputing the associated POS over all sensors and time points (*n* = 1000). We then built a null distribution at the group level by randomly picking one sample per individual from the previous distributions, averaging the POS data points over individuals and repeating these operations (*n* = 10,000). A spatiotemporal clustering procedure was then performed on the POS index of interest based on ILL and no-ILL conditions, using the null group distribution. In detail, this procedure first thresholded the group-level POS based on the group-level null distribution (the 2.5 and 97.5 percentiles were used as criteria, corresponding to a two-sided test at α = 0.05). These thresholded data points were then clustered on the basis of spatial and temporal adjacency (minimal cluster size, 10 data points). The statistic of each cluster was calculated by taking the sum of the POS values within this cluster. The cluster statistics were finally thresholded at an α-level of 0.05, using a distribution obtained by performing the same clustering procedure for each permutation of the null distribution and by taking the maximum cluster statistics of the resulting clusters. The cluster *p* value therefore corresponds to the probability of a value from this distribution to be above or equal to the cluster statistic.

The POS index of interest (see Fig. 3*A*,*B*, plots) was *z*-scored relative to the mean and SD of the group-level null distribution. This analysis was conducted separately for the alpha and theta bands, and for each type of sensor. The POS values on planar gradiometers were combined using the Euclidean norm before performing the spatiotemporal clustering procedure.

#### Source reconstruction

To estimate the likely generators of the effect in sensors space, we proceeded with source reconstruction. The anatomic MRI were imported with BrainVisa and segmented with the FreeSurfer image analysis suite. A three-layer boundary element model (BEM) surface was generated to constrain the forward model. Individual forward solutions or head models (10,242 icosahedrons/hemisphere, 3.1 mm spacing) were computed using the individual BEM model constrained by the anatomic MRI. The anatomic MRI and MEG sensors were manually realigned with a coregistration procedure based on digitalized anatomic landmarks using MRiLab (http://mrilab.sourceforge.net) and manually fine-tuned with the MNE-Python suite (Gramfort et al., 2014). The noise covariance matrix was estimated from an individual’s 5 min resting state recorded at the beginning of the session. The inverse operator (lead-field matrix) was computed from the noise covariance matrix and the forward model. The dSPM method was used to create the source estimates using a loose orientation constraint (loose = 0.4, depth = 0.8). The source estimates were then morphed into the common Freesurfer average brain for subsequent group analysis.

To estimate the generators underlying the statistical effects observed in the sensor data, alpha power was also computed in source space for each time point with Morlet wavelets (8–12 Hz, 6.7 cycles). Then we correlated the individual difference in alpha power between ILL and no-ILL trials with the individual percentage of illusory perception. The Spearman’s correlation was obtained for each voxel and time point. For determining anatomic locations, we used the aparc.a2009s parcellation (Destrieux et al., 2010) and selected the labels containing at least 25 significant voxels at *p* < 0.01, during at least one time point included in the significant temporal window found from the sensor analysis. This analysis has not been corrected for multiple comparisons as it is only intended to visualize significant effects in the sensors. We used a similar procedure for the visualization of POS sources. We computed individual alpha POS between ILL and no-ILL trials, averaged across participants, and selected labels containing at least 25 voxels with a POS value above the 99th percentile value (corresponding to *p* = 0.01) from the null group-level distribution computed in the sensor analysis.

### Data availability

Analysis code will be made available on request.

## Results

### Behavioral results

Participants (*N* = 13) were presented with a sequence of three flashes and were asked to determine the location of the second flash in a 2-AFC task. In test trials, the second flash was presented at the same location as the first flash; in control trials, the second flash was presented at an intermediate spatial location between the first and last flash (Fig. 1*A*). In both test and control trials, the three-flash sequences were always presented in the right visual periphery, either going from left to right (outward) or from right to left (inward; Fig. 1*B*). In the control condition, participants correctly reported the location of the second flash in 81% (SD = 1) of the trials (Fig. 1*C*). In the test condition, the sequences were designed to elicit 50% of illusory responses, and, on average, participants reported an illusory displacement of the second flash in 51% (SD = 5; *t* test against 50%, *p* = 0.848; BF = 0.21) of the trials (Fig. 1*C*). A two-way repeated-measures ANOVA was conducted on the probability of “intermediary position” responses with factors of condition (two: test, control) and direction (two: inward, outward), and participants as a random effect. We found a main effect of experimental condition (*F*_(1,12)_ = 37.94, *p* = 5.10^−5^, η^2^_p_ = 0.76) showing that participants reported the second flash at an intermediate position significantly more often in control than in test trials, which was expected by the experimental design. We found no significant effects of sequence direction (*F*_(1,12)_ = 1.83, *p* = 0.201, η^2^_p_ = 0.13) and no interactions between condition and sequence direction (*F*_(1,12)_ = 1.59, *p* = 0.231, η^2^_p_ = 0.12). Noticeably, the interindividual variability in the fraction of illusory perceived trials was large (range = 12–95%) and the individual percentages of illusory perception did not correlate between sequence directions (Spearman correlation: *R* = 0.37, *p* = 0.217, BF = 0.74; Fig. 1*C*, right).

To capture individual differences in internal priors, we used the following two measures: the percentage of illusory perception and the criterion as defined in signal detection theory. We calculated these two behavioral metrics for each individual and each sequence direction (Fig. 1*C*,*D*). Most participants showed a liberal criterion (*c* < 0), indicating that they were biased toward reporting the illusion. To ensure that interindividual differences in illusory perception did not reflect a general effect of performance rather than a difference in individuals’ internal priors, we correlated the percentage of correct responses between test trials and control trials. A positive correlation would favor the hypothesis that interindividual differences in test trials comes from a general effect of performance. Instead, we found a negative correlation in the inward condition (*R* = −0.60, *p* = 0.032, BF = 3.44) and a trend toward a negative correlation in the outward condition (*R* = −0.51, *p* = 0.078, BF = 2.88); participants perceiving the illusion more often were better in the control task. In other words, participants showing a tendency to perceive the stimulus at an intermediary position did so in both test and control trials, independently of the actual location of the stimulus. Consistent with this, we also found a strong correlation between an individual’s percentage of illusory responses with the individual’s criteria for both directions (outward: *R* = −0.91, *p* < 2.10^−16^, BF = 706; inward: *R* = −0.92, *p* < 2.10^−16^, BF = 1024). This result suggests that the percentage of illusion reported by an individual may reflect biases induced by internal priors. We discuss further the conceptual and computational differences between perceptual and decisional biases, prior expectations, and these two metrics in the Discussion section.

We also investigated participants’ RTs, which can provide insights into the chronometry of the spatial reorganization in the rabbit illusion. We performed a two-way repeated-measures ANOVA on log-transformed RTs with factors of sequence direction (two: inward, outward) and perceptual report (three: illusion, no-illusion, control) with participants as random effect (Fig. 1*E*). We found a main effect of perceptual report (*F*_(2,12)_ = 6.750, *p* = 0.004, η^2^_p_ = 0.36) and a marginal effect of sequence direction (*F*_(1,12)_ = 4.241, *p* = 0.062, η^2^_p_ = 0.26). We found no interactions between both factors (*F*_(2,24)_ = 1.369, *p* = 0.274, η^2^_p_ = 0.10). A *post hoc* Bonferroni-corrected paired *t* test showed that response times in control trials were on average 130 ms faster than in illusion trials (*p* = 10^−4^, *t* = 4.88, BF = 493.0) and 130 ms faster than in no-illusion trials (*p* = 0.004, *t* = 3.61, BF = 26.7). Slower response times in the test as compared with the control trials could be because of an additional perceptual or decisional process required to deal with the increased uncertainty of perceptual outputs in the test condition. Although the effect of sequence was marginally significant in the ANOVA, response times averaged across all trials were also 71 ms faster in the outward than in the inward condition (*p* = 0.014, *t* = 2.59, BF = 3.1), consistent with previous studies using looming/receding stimuli (Cappe et al., 2009; Tyll et al., 2013).

### Prestimulus alpha power correlates with individual tendencies to perceive the illusion

Our working hypothesis was that alpha power could reflect the influence of an internal prior favoring constant-speed motion. We used two behavioral measures as a proxy for this prior: the individual percentage of illusory trials, as a strong prior should increase the overall probability to perceive the illusion, and the criterion, a metric used in signal detection theory to quantify decisional biases.

We expected that alpha fluctuations were predictive of whether participants would follow their tendency to perceive the illusion or not. We thus performed a Spearman correlation analysis between the individual likelihood of perceiving the illusion, or the individual criterion, and the alpha power difference between illusion and no-illusion trials for each type of sensor. We performed this analysis separately on each direction because the percentage of illusory perception and the criterion depended on the sequence (Fig. 1*C*, right, *D*).

**Figure 2.**
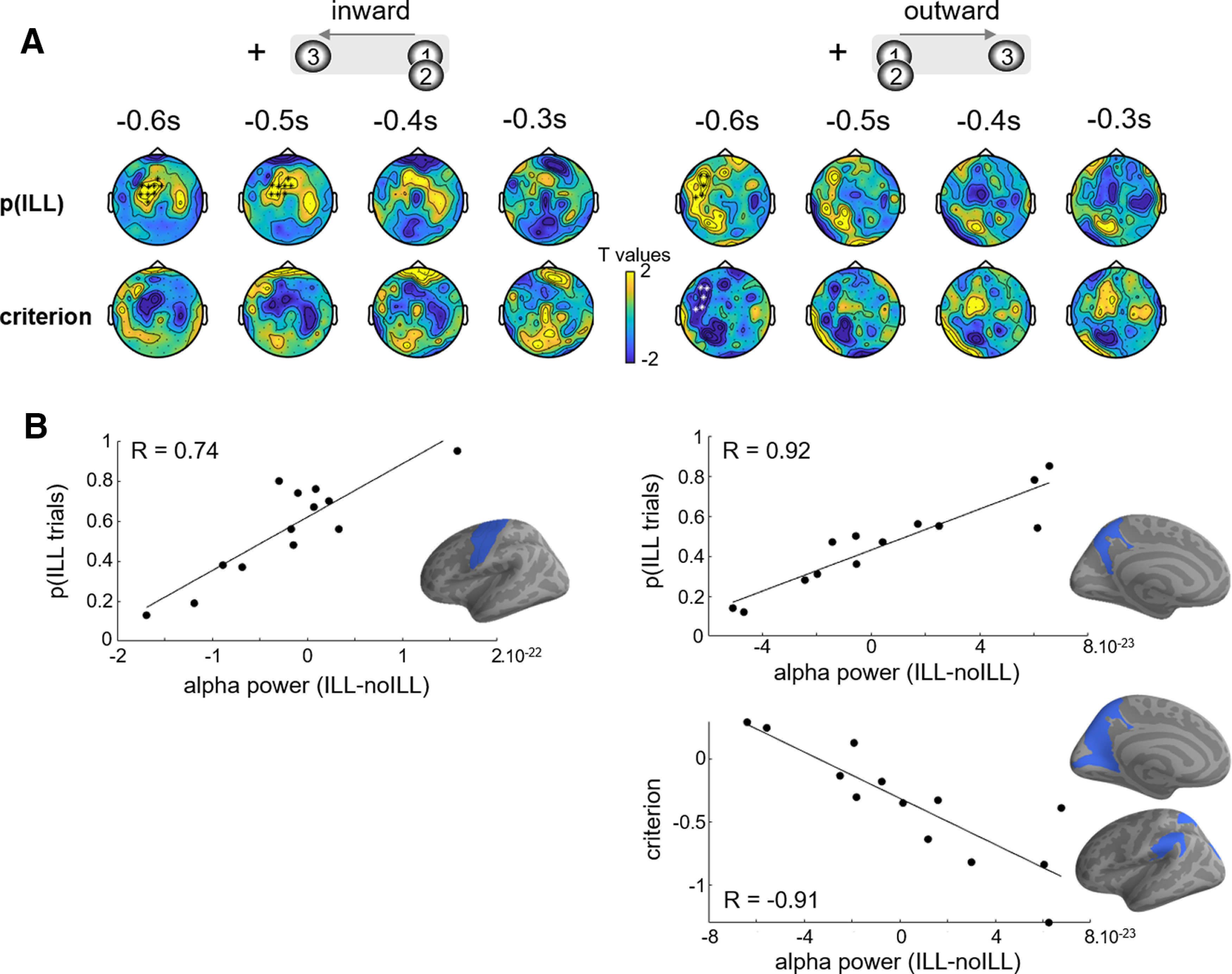
Prestimulus alpha power fluctuations correlate with the individual tendency to perceive the illusion. ***A***, Spatiotemporal clustering on the between-individual correlation between the prestimulus alpha difference in ILL–no-ILL for inward (left) and outward (right) conditions and the probability of perceiving the illusion (top) or the individual criterion (bottom). Only the gradiometers are plotted, the significant sensors are highlighted in black or white. ***B***, The correlation is plotted for significant time points and sensors. Source reconstruction of the correlation between the prestimulus alpha difference in ILL–no-ILL for inward (left) and outward (right inset) conditions, and the probability of perceiving the illusion (top) or the criterion (bottom). Blue labels indicate that at least 25 voxels show a significant correlation (*p* < 0.01) within the significant temporal window found in the sensor analysis.

Using a spatiotemporal cluster-based permutation test, we found a significant effect between the alpha power and the percentage of illusory trials for both sequence directions in left central gradiometers (Fig. 2*A*; inward: −603 to −513 ms, *T*_sum_ (cluster statistic) = 234.65, *p* = 0.029; outward: −585 to −513 ms, *T*_sum_ = 148.97, *p* = 0.034). The correlation coefficient for the significant sensors and time points was 0.74 (bootstrap-based 95% CI = 0.21, 0.96; BF = 451) and 0.92 (bootstrap-based 95% CI = 0.70, 0.99; BF =3855) for the inward and the outward conditions, respectively (Fig. 2*B*). No significant differences were found in magnetometers or EEG sensors. Using the same analysis, we found a significant correlation between alpha power in gradiometers and criterion in the outward condition only (Fig. 2*A*: −603 to −522 ms, *T*_sum_ = −186.30, *p* = 0.028). The correlation coefficient for the significant sensors and time points was −0.91 (bootstrap-based 95% CI = −1, −0.65; BF = 171). The sensor topography of the *t* values shown in Figure 2*A* is very similar to that observed for the correlation with the percentage of illusion, which is expected because of the strong correlation between these two metrics.

We then investigated further whether the correlation between alpha power and the percentage of illusion occurred in similar sensors and time points for both directions. Although the effect emerged in overlapping time windows, the significant differences in gradiometers were spatially distinct between directions. As the spatiotemporal clustering does not specifically address the localization of an effect, we computed the correlation for one direction within the cluster of the other direction. The correlations were not significant (inward data in outward cluster: *R* = −0.43; *p* = 0.140; bootstrap-based 95% CI = −0.87, 0.14; outward data in inward cluster: *R* = 0.07; *p* = 0.809; bootstrap-based 95% CI = −0.50, 0.59). Bayes factors indicated anecdotal evidence for a correlation in one condition, and substantial evidence for no correlation in the other one (inward data in outward cluster, BF = 1.739; outward data in inward cluster, BF = 0.229). Additionally, we performed a conjunction analysis based on the assumption of common location across all time points and sensors, which did not yield any significant effect (spatiotemporal clustering, *p* < 0.05). Altogether, these analyses suggested that the correlation arises from distinct sensors for each direction.

Finally, we addressed the specificity of the effect by performing the same correlation analysis on theta, beta, low-gamma, and high-gamma frequency bands. No significant difference (*p* < 0.05) was found for any of the sensor types, suggesting that the correlations between brain activity and the tendency to perceive the illusion or the criterion may be specific to the alpha band.

To estimate the sources underlying the significant correlations at the sensor level, we computed the alpha power difference between illusion and no-illusion for each voxel. For each sequence direction, we averaged this difference across the significant time windows and correlated it with individual percentages of illusion. Note that this analysis is not independent of the sensor-level analysis as it is intended to illustrate the latter. The insert in Figure 2*B* shows the atlas regions containing at least 25 voxels with a significant correlation at *p* < 0.01. The left precuneus was found for the outward condition (31 significant voxels; *R* = 0.75 ± 0.04, mean ± SD), and the left precentral gyrus (34 significant voxels; *R* = 0.75 ± 0.04), left central superior sulcus (55 significant voxels; *R* = 0.75 ± 0.04), and precentral superior sulcus (48 significant voxels; *R* = 0.75 ± 0.04) were found for the inward condition. We performed the same analysis with individual criteria in the outward condition, and found the left precuneus (76 significant voxels; *R* = −0.81 ± 0.07), the left parietal inferior gyrus (37 significant voxels; *R* = −0.82 ± 0.06), the left parietal superior gyrus (31 significant voxels; *R* = −0.75 ± 0.04), the left calcarine sulcus (32 significant voxels; *R* = −0.75 ± 0.05), the left occipital superior and transversal sulci (48 significant voxels; *R* = −0.79 ± 0.05), and the left parieto-occipital sulcus (79 significant voxels; *R* = −0.80 ± 0.07; Fig. 2*B*, bottom).

Last, we tested the alternative working hypothesis that prestimulus alpha power directly predicts the perception of the illusion. According to this hypothesis, high alpha power would be associated with illusory percepts, regardless of the individual’s prior. Indeed, previous literature has shown a link between alpha power and illusory perception, even if the direction of the effect depends on the tested illusion (Lange et al., 2014). To test this, we contrasted the power of alpha preceding illusion and no-illusion trials, for combined inward and outward sequences. No significant differences (*p* < 0.05) were found after spatiotemporal clustering *t* test for any of the sensor types. We additionally computed the Bayes factor for each sensor and time point and found that 46% of sensors and time points showed positive to substantial evidence in favor of the null hypothesis (BF < 0.33), while only 0.6% of sensors and time points showed positive to substantial evidence for a significant difference between illusion and no-illusion trials (BF > 3).

In summary, we found that prestimulus alpha power can predict whether an individual will follow or go against his/her tendency to perceive the illusion, not the illusion per se. Additionally, the localization of this effect may depend on the direction of the sequence.

### Prestimulus alpha phase predicts the perception of the illusion

**Figure 3.**
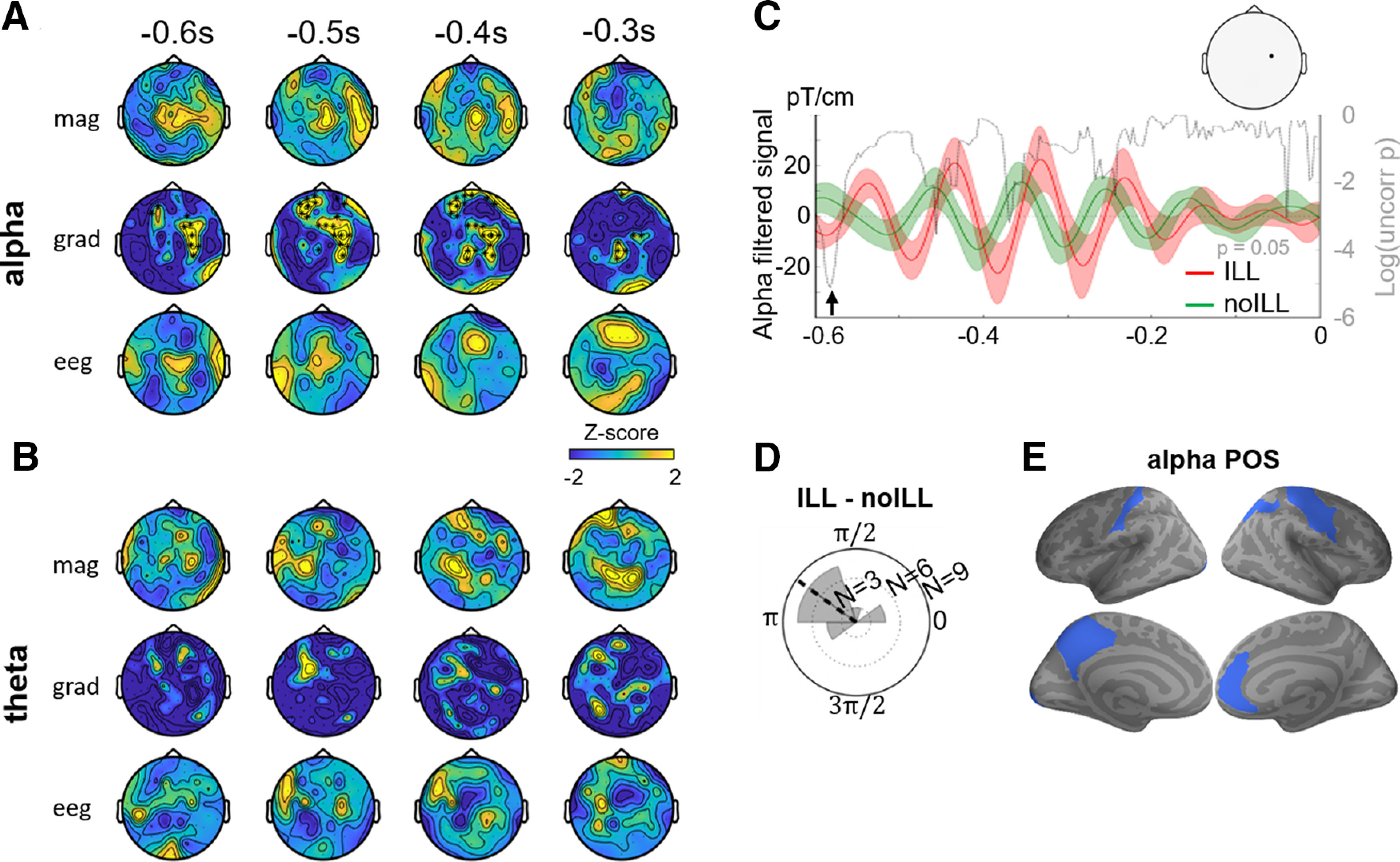
Alpha phase predicts the perception of the illusion. ***A***, We investigated whether prestimulus alpha phase predicts the perception of the illusion for combined sequence directions, using POS. A significant difference was found in the gradiometers. Each topomap shows a *z*-scored POS averaged over a 100 ms time window. ***B***, No significant differences were found when the theta POS was tested during the presequence period. ***C***, For visualization, we selected the channel corresponding to maximal *z*-scored POS (left topoplot) and filtered the signal in the alpha band, for each response (ILL, red; no-ILL, green). Shaded areas show SEM. The phase difference between ILL and no-ILL signals was assessed by a Rayleigh test for each time point. The log of the obtained *p* values is plotted in dashed gray line (horizontal dotted line corresponds to *p* = 0.05). ***D***, We selected the time point corresponding to the minimal *p* value (***B***, arrow) and plotted the circular histogram of phase difference between ILL and no-ILL (mean angle shown by the dashed line). ***E***, Source reconstruction of the POS between ILL and no-ILL trials and the prestimulus alpha difference ILL–no-ILL, with blue labels indicating that at least 25 voxels have a POS value corresponding to *p* = 0.01 within the significant temporal window found in the sensor analysis.

Our last hypothesis was that the phase of prestimulus oscillations could dissociate illusion trials from no-illusion trials. Based on the literature, we tested two potentially involved frequencies, theta (4–7 Hz) and alpha (8–12 Hz). First, we estimated for each frequency the phase consistency across trials during the prestimulus period using the phase opposition index (i.e., POS; see Materials and Methods). A significantly positive POS index indicates that the phase of oscillatory activity concentrates around distinct preferred angles for each condition. We ran a spatiotemporal cluster-based test based on a surrogate null distribution to assess the statistical significance of POS between the illusion and no-illusion trials on combined inward and outward sequences (Fig. 3*A*). We found a significant difference in the alpha band in gradiometers (−594 to −216 ms, *T*_sum_ = 31.71, *p* = 0.033) but not in magnetometers or in EEG sensors. No significant differences were found in the theta band (Fig. 3*B*).

To visualize this effect, we selected the sensor corresponding to the maximal *z*-scored POS within the significant sensors, and plotted the grand average alpha-filtered signal for each response (Fig. 3*B*). We then computed the instantaneous single-trial phase in the alpha range for this sensor, each response, and each participant. We confirmed on this selected electrode the significance of the phase difference between the illusion and the no-illusion trials using a Rayleigh test on each time point. The uncorrected *p* values shown in Figure 3*C* (gray curve) reveal a minimal *p* value at −582 ms (*p* = 0.006). The histogram of the phase differences across individuals at that time point showed a mean phase angle of −144° (Fig. 3*D*), suggesting that perceiving the illusion and not perceiving the illusion can be predicted by opposing alpha phases before the presentation of the visual sequence.

We used source reconstruction and localized the significant difference in sensors by computing the POS between illusion and no-illusion trials for each voxel and each time point. Figure 3*E* shows the labels containing at least 25 voxels with a POS value above a threshold corresponding to *p* = 0.01 (see Materials and Methods). This revealed significant voxels in the left precuneus (precuneus, 25 voxels; cingulate sulcus, 53 voxels; subparietal sulcus, 28 voxels), left occipital pole (33 voxels) as well as left somatosensory area (postcentral cortex, 29 voxels), right somatosensory area (postcentral, 34 voxels; precentral, 30 voxels; central cortex, 35 voxels), right intraparietal sulcus (28 voxels), and right anterior cingular cortex (43 voxels).

## Discussion

We investigated the role of prestimulus oscillatory activity in visual postdiction, which we operationally defined as the mislocalization of an event following the temporal structure of the sequence in which it is embedded (Dennett and Kinsbourne, 1992; Shimojo, 2014). This illusion might be explained by the influence of an internal prior favoring the perception of slowly moving objects at constant speed (Goldreich and Tong, 2013). The interindividual variability in perceiving the illusion and in the response bias (criterion) measured the impact of such priors on behavior, which were found to correlate with fluctuations of frontoparietal alpha power. Additionally, the phase of prestimulus alpha, but not its power, differed between illusion and no-illusion reports.

### Prestimulus alpha power in frontoparietal areas relates to internal priors in postdiction

Based on the assumption that postdiction is driven by prior expectations, and on previous literature indicating that the influence of priors on perception is reflected in low-frequency oscillatory activity (Mayer et al., 2016; Sherman et al., 2016; Rungratsameetaweemana et al., 2018; Samaha et al., 2018), we hypothesized that perceptual reports related to the rabbit illusion may also rely on fluctuations of prestimulus activity.

We investigated prestimulus alpha activity through the prism of predictive coding, with alpha oscillations implicated in perceptual decision-making (Bauer et al., 2014; Alamia and VanRullen, 2019) and in the generation of internal predictions (Sanders et al., 2014). At the population level, alpha power has been shown to correlate with decision criteria rather than task accuracy (Limbach and Corballis, 2016; Craddock et al., 2017; Iemi et al., 2017; Samaha et al., 2017; Iemi and Busch, 2018). At the individual level, the influence of such priors on behavior manifests in terms of a significant relationship between alpha power and the individual’s idiosyncratic biases on perceptual timing (Grabot et al., 2017; Grabot and Kayser, 2020).

Consistent with recent studies (Limbach and Corballis, 2016; Iemi et al., 2017; Samaha et al., 2017), we found that alpha power was not related to the serial and objective ordering of the sequence, but rather reflected the tendency of an individual to perceive the rabbit illusion. Our results suggest that, for a participant with a favorable bias toward the illusion, reporting the illusory percept will be associated with a higher power of prestimulus frontoparietal alpha. Conversely, for a participant with a bias toward veridical perception, reporting the illusory percept will be associated with a lower prestimulus alpha power. In short, a higher prestimulus alpha power predicts the perception of a sequence following the individual’s bias, whereas a lower alpha power predicts the perception of a sequence opposite to the individual’s bias. We found a similar correlation with alpha power fluctuations using the individual’s criterion. This indicates that an increase in prestimulus alpha power is followed by the perception of the illusion for participants with a more liberal criterion (i.e., more likely to report the target at the intermediate position). However, this correlation was found only for outward sequences.

While the two metrics used to capture an individual’s internal bias (ratio of perceived illusion and criterion) are related by definition, a conceptual difference regarding their relationship with internal priors may explain this discrepancy. The constant-speed prior should mainly bias the percentage of perceived illusion in the test but not in the control trials: a constant-speed prior agrees with sensory evidence in control subjects since stimuli are isochronous and equidistant. In control trials, the ratio of correct to incorrect responses should therefore be marginally influenced by the internal prior. To the contrary, this prior disagrees with evidence of isochronous but nonequidistant test sequences, thereby possibly biasing participants’ responses. This explains why the percentage of illusory responses may be a more conservative measure of the impact of a prior on perception when compared with the decision criterion, which is affected by additional response biases.

The cortical sources of prestimulus effects were estimated in parietal and frontal regions, in agreement with the involvement of top-down signals carried by low-frequency oscillations (Mayer et al., 2016; Michalareas et al., 2016). The precuneus, found in the outward condition, is generally related to visuospatial imagery (Cavanna and Trimble, 2006), but also to the maintenance of past stimulus history during multisensory integration (Park and Kayser, 2019). During bistable perception, previous work has shown that the maintenance of a current perceptual representation engages frontal and parietal areas (Kanai et al., 2010; Vernet et al., 2015; Piantoni et al., 2017). Interestingly, a stronger alpha power is associated with longer perception of a bistable stimulus (Piantoni et al., 2017), and prestimulus feedback connectivity in the alpha range can predict which percept is favored (Rassi et al., 2019). In line with previous work, additional regions found for the inward condition included the motor and premotor cortices, known to play a central role in timing, especially in the momentary indexing of ongoing time (Macar et al., 2006; Coull et al., 2011). The observed prestimulus alpha activity correlating with individual biases was also located in frontal areas, possibly related to cognitive control (Grabot and Kayser, 2020). More generally, these findings converge in conferring on alpha power a role in the stability of internal states, consistent with our observations that prestimulus frontoparietal alpha power may control the influence of internal models that bias perception.

The differences in the topographies of the alpha effects between the inward and the outward conditions remain to be explained. One possibility is that the analysis was underpowered, given the rather small sample size, and hence did not detect all the involved cortical regions across both conditions. However, it could also be that the relevant constant-speed priors involved in postdiction differ between movement directions and recruit different brain regions: looming stimuli are processed differently compared with the receding stimulus in timing and space likely because of their potential ecological importance (van Wassenhove et al., 2008; Hubbard and Neuhoff, 2018). Future work could specifically focus on distinguishing the neural processing of inward and outward movements.

### Prestimulus alpha phase predicts illusory reports

A major working hypothesis for the role of low-frequency oscillations is that they segment neural processing into discrete cognitive units (Lakatos et al., 2005). Alpha phase has been shown to influence the perception of an upcoming stimulus (Busch et al., 2009; Mathewson et al., 2009) and is related to cortical excitability (Haegens et al., 2011; Kayser et al., 2015). These empirical studies have led to the idea that visual information is sampled rhythmically at alpha frequency (VanRullen and Koch, 2003; Bonnefond and Jensen, 2012; Samaha and Postle, 2015; VanRullen, 2016b). Importantly, the relation between alpha phase and perception has mostly been studied using a single stimulus. Here, we used a three-flash sequence with a perceptual report concerning the second stimulus and found that the alpha phase preceding the first flash influenced the perceived location of the second flash.

We can speculate about several possible mechanisms underlying the impact of prestimulus alpha activity on the perception of a sequence and rule out some others. Low-frequency alpha and theta cycles have been proposed to act as perceptual integration windows (Cecere et al., 2015; Milton and Pleydell-Pearce, 2016; Wutz et al., 2016, 2018; Ronconi et al., 2017; Rohe et al., 2019). In the sequences used here, flashes were separated by the equivalent of two to three alpha cycles (188 or 272 ms), and this prevented any two visual stimuli from occurring within the same alpha cycle. We also did not find significant results for theta phase, whose period would match that of the visual sequence used here. Although we cannot conclude that there is an absence of an effect, this may be explained by the fact that theta phase is involved in spatial attention (Helfrich et al., 2018), which is not at the core of the postdictive process, although it can modulate it (Kilgard and Merzenich, 1995). Temporal integration (or binding) and encoding windows refer to the period of time during which sensory inputs are fused (Theunissen and Miller, 1995; van Wassenhove et al., 2007) or the period of time needed to encode relevant information (Panzeri et al., 2010; van Wassenhove, 2013), respectively. In both cases, these integration/encoding windows imply that temporal information is lost (van Wassenhove et al., 2007; van Wassenhove, 2013; Melcher et al., 2014). Yet, this is not the case in the rabbit illusion considering that the sequences of three flashes are reported. Hence, the rabbit illusion requires the coding of all three sensory events and their temporal relationships. One can therefore consider alpha cycles as processing (rather than integration) windows, whose informational content can be revised during the next alpha cycle. This idea fits with the multiple-draft theory, positing that conscious content results from the cyclic competition between different representations (Dennett and Kinsbourne, 1992).

A last and nonexclusive mechanism could be that the prestimulus alpha phase only influences the processing of the first flash, since the first stimulus is likely to reset the phase of the ongoing neural oscillations (Romei et al., 2012; Canavier, 2015). Since the phase of prestimulus alpha was found to improve or impair the detection of a visual stimulus (Busch et al., 2009; Mathewson et al., 2009), a favorable alpha phase could improve the processing of the first stimulus, therefore decreasing the uncertainty on the temporal information of the full sequence. This temporal information would then combine with the constant slow speed before increasing the probability of perceiving equidistant stimuli. In sum, future studies are needed to determine the specific influence of alpha phase on the perception of sequences and temporally extended stimuli.

Regarding the localization of this phase effect, we found implicated regions in occipital cortex, typically observed in studies investigating phase effects on stimulus detection (Mathewson et al., 2009; Sherman et al., 2016). In a previous study, the prestimulus alpha phase in occipital areas correlated with trial-by-trial variability of the flash-lag effect, another illusion possibly relying on postdictive processes (Chakravarthi and VanRullen, 2012). We noticed that the left precuneus and left premotor areas were implicated in both the alpha power/prior and the alpha phase/accuracy effects, suggesting that they play a key role in postdiction. One fMRI study investigating postdiction also found the implication of premotor and prefrontal cortex, although they used a tactile version of the rabbit illusion (Blankenburg et al., 2006). These premotor and prefrontal areas could be involved in general processes underlying postdiction, while the precuneus activity would be specific to visual modality.

Altogether, this study attempts to fill an important gap in the understanding of postdiction, an exquisite temporal phenomenon whose mechanisms are highly debated (Shimojo, 2014). Postdictive phenomena may rely on retroactive processes akin to perceptual reconstruction. Unlike prediction, postdiction was intuited to occur after the presentation of the sequence of stimuli. This theoretical argument is at odds with our findings: the initial states of nonlinear dynamics (alpha power and phase) can partly determine the perceptual outcome of the rabbit illusion before the presentation of the visual sequence has taken place. Postdictive phenomena may be a special case of predictive mechanisms (Hogendoorn and Burkitt, 2019) in which strong individual priors may shape subjective perception.

## Synthesis

Reviewing Editor: Christina Zelano, Northwestern University Decisions are customarily a result of the Reviewing Editor and the peer reviewers coming together and discussing their recommendations until a consensus is reached. When revisions are invited, a fact-based synthesis statement explaining their decision and outlining what is needed to prepare a revision will be listed below. The following reviewer(s) agreed to reveal their identity: Vincenzo Romei, Andreas Wutz.

The reviewers agreed that the manuscript is much improved. One reviewer had a few remaining concerns that are critical to address, including lack of methodological and statistical details, and a conceptual question. This information needs to be added to the manuscript. Please see detailed comments from this reviewer below:

The paper has clearly improved compared to the first submission. In fact, the authors have done a convincing job addressing most of my earlier comments and concerns. I particularly appreciate their added analyses with respect to the response criterion, which supports their point about the differential roles of alpha power and phase on bias vs. accuracy, respectively. Moreover, they have re-structured many sections and added previously missing infos. At large, this has led to a much clearer and more concise report of their findings.

However, I do have two more points (one methodological, one conceptual in nature), which I find insufficiently addressed and hence require further elaboration.

1) conceptual issue

The authors now discuss their findings in the context of the multiple-drafts theory by Dennett & Kinsbourne (1992), which is the core theory underlying postdiction. They claim that their interpretation of the results in terms of processing windows (rather than integration windows) are consistent with this theory. In this context, I have a theoretical question. The multiple-drafts theory posits that different aspects of our sensory environment are computed on different time scales and in parallel. Of course, these “multiple drafts” of perpetual reality need to be combined and integrated at some point. We don’t perceive multiple visual realities at the same time, right? Why is then an interpretation of oscillatory cycles in terms of processing windows (rather than integration windows) more fitting here?

In this context, you mention the work of Wutz et al., 2016, 2018 as paramount examples for integration windows in your response letter, but you do not cite this work in your paper. Is there a reason for that?

2) methods issue

I still don’t fully understand all the details about how cluster-based permutations were used to correct for multiple comparisons for the POS analysis. I guess, you will need to walk me through this procedure step-by-step. Let’s see up to which point I can still follow:

1. You compute the observed POS for each participant and then average the POS metrics over participants to get the observed group-level POS.

2. You randomly assign trials to either pseudo-Illusion / pseudo-no Illusion trial-subsets, compute POS and do this multiple times to generate random permutation distributions of POS for each participant.

3. You draw from each subject-specific permutation distribution of POS, average them over subjects and do this multiple times to generate random permutation distributions of group-level POS.

4. From here you look up whether your observed group-level POS is bigger than the 97.5% quantile of the random permutation distributions of group-level POS. This gets you a p-value for each sensor and time point.

Up to here the procedure would be indeed identical to the one described in Busch et al., J Neuro, 2009. However, from here I fail to understand how cluster statistics (both observed and permuted) are computed. Back then, Busch et al. (2009) used the FDR procedure to correct for multiple comparisons. How were spatial-temporal clusters built then?

Also, I have seen that you report summed T-values (p. 20) and actual t-values (Figure 3), in order to support your POS effects. Where do those come from?

minor:

The authors still use the phrase “significant clusters", which is methodologically incorrect (lines 238 / 239 and 244/245 and 785/786).

## Author Response

Dear Editors,

We are grateful for your overall positive feedback and the two additional comments. We addressed the methodological issue by clarifying the statistical analysis used in the phase opposition analysis, and the conceptual issue by explicitly defining the role of integration versus processing windows into the multiple-draft theory.

Please find below a point-by-point reply.

The reviewers agreed that the manuscript is much improved. One reviewer had a few remaining concerns that are critical to address, including lack of methodological and statistical details, and a conceptual question. This information needs to be added to the manuscript. Please see detailed comments from this reviewer below:

The paper has clearly improved compared to the first submission. In fact, the authors have done a convincing job addressing most of my earlier comments and concerns. I particularly appreciate their added analyses with respect to the response criterion, which supports their point about the differential roles of alpha power and phase on bias vs. accuracy, respectively. Moreover, they have re-structured many sections and added previously missing infos. At large, this has led to a much clearer and more concise report of their findings.

However, I do have two more points (one methodological, one conceptual in nature), which I find insufficiently addressed and hence require further elaboration.

1) conceptual issue

The authors now discuss their findings in the context of the multiple-drafts theory by Dennett & Kinsbourne (1992), which is the core theory underlying postdiction. They claim that their interpretation of the results in terms of processing windows (rather than integration windows) are consistent with this theory. In this context, I have a theoretical question. The multiple-drafts theory posits that different aspects of our sensory environment are computed on different time scales and in parallel. Of course, these “multiple drafts” of perpetual reality need to be combined and integrated at some point. We don’t perceive multiple visual realities at the same time, right? Why is then an interpretation of oscillatory cycles in terms of processing windows (rather than integration windows) more fitting here?

A temporal window of integration is generally defined as the period of time during which sensory inputs from events occurring in that period are fused, implying that the temporal information is lost (Melcher et al., 2014, Procedia; van Wassenhove, Grant & Poeppel, 2007, Neuropsychologia). A processing window would be defined in a looser sense as a period of time during which information is processed. In the rabbit illusion, the temporal information of events is not lost (on the contrary, it is hypothesized to inform where visual events will be perceived) so that’s why we believe it may be more relevant to speak about processing rather than integration windows. That said, we agree with the Reviewer that, according to the multiple-draft theory, these temporal windows are potentially integrated over longer time scales, to lead to a final percept.

We have clarified the Discussion section (p. 25).

In this context, you mention the work of Wutz et al., 2016, 2018 as paramount examples for integration windows in your response letter, but you do not cite this work in your paper. Is there a reason for that?

None, they are now cited (p. 24).

2) methods issue

I still don't fully understand all the details about how cluster-based permutations were used to correct for multiple comparisons for the POS analysis. I guess, you will need to walk me through this procedure step-by-step. Let's see up to which point I can still follow:

1. You compute the observed POS for each participant and then average the POS metrics over participants to get the observed group-level POS.

2. You randomly assign trials to either pseudo-Illusion / pseudo-no Illusion trial-subsets, compute POS and do this multiple times to generate random permutation distributions of POS for each participant.

3. You draw from each subject-specific permutation distribution of POS, average them over subjects and do this multiple times to generate random permutation distributions of group-level POS.

4. From here you look up whether your observed group-level POS is bigger than the 97.5% quantile of the random permutation distributions of group-level POS. This gets you a p-value for each sensor and time point.

Up to here the procedure would be indeed identical to the one described in Busch et al., J Neuro, 2009. However, from here I fail to understand how cluster statistics (both observed and permuted) are computed. Back then, Busch et al. (2009) used the FDR procedure to correct for multiple comparisons. How were spatial-temporal clusters built then?

The Reviewer is correct regarding these 4 steps. Indeed, to correct for multiple comparisons, we did not use the FDR procedure used in Busch et al., 2009. Instead, we used a spatio-temporal clustering approach (based on the Matlab function spm_bwlabel), which has the advantage of taking into account the spatio-temporal correlation structure of the data (Maris & Oostenveld, 2007).

In detail, this procedure first thresholded the group-level POS based on the group-level null distribution (using the 97.5 percentile as a criterion). Then, these thresholded data points are clustered on the basis of spatial and temporal adjacency (minimal cluster size = 10 data points). The statistic of each cluster is calculated by taking the sum of the t-values within this cluster. The clusters statistics are finally thresholded at an alpha-level of 0.05.

We have now clarified the Methods section (p. 13).

Also, I have seen that you report summed T-values (p. 20) and actual t-values (Figure 3), in order to support your POS effects. Where do those come from?

The summed t-values (p. 20) correspond to the cluster-level statistics of the reported clusters. The t-values plotted in Fig. 3A and 3B are calculated, for each sensor and each time point, as follows:

tval = (POS-m)/s

with POS the group-level POS, m the mean POS over the group-level null distribution and s the standard deviation of the group-level null distribution. We added this information in the manuscript (p. 13).

minor:

The authors still use the phrase “significant clusters", which is methodologically incorrect (lines 238 / 239 and 244/245 and 785/786).

This is now corrected.
